# Cardiac Function after Modern Radiation Therapy with Volumetric Modulated Arc Therapy or Helical Tomotherapy for Advanced Left-Breast Cancer Receiving Regional Nodal Irradiation

**DOI:** 10.3390/bioengineering9050213

**Published:** 2022-05-16

**Authors:** Pei-Yu Hou, Chen-Hsi Hsieh, Le-Jung Wu, Chen-Xiong Hsu, Deng-Yu Kuo, Yueh-Feng Lu, Yen-Wen Wu, Hui-Ju Tien, Shih-Ming Hsu, Pei-Wei Shueng

**Affiliations:** 1 Division of Radiation Oncology, Department of Radiology, Far Eastern Memorial Hospital, New Taipei City 220, Taiwan; jcgv03.be07@nycu.edu.tw (P.-Y.H.); chenciab@gmail.com (C.-H.H.); lejung.tw@yahoo.com.tw (L.-J.W.); cxhsu@mail.femh.org.tw (C.-X.H.); dykuo@mail.femh.org.tw (D.-Y.K.); charmeur0601@yahoo.com.tw (Y.-F.L.); catju@mail.femh.org.tw (H.-J.T.); 2 Department of Biomedical Imaging and Radiological Sciences, National Yang Ming Chiao Tung University, Taipei 112, Taiwan; 3 School of Medicine, College of Medicine, National Yang Ming Chiao Tung University, Taipei 112, Taiwan; wuyw0502@gmail.com; 4 Institute of Traditional Medicine, School of Medicine, National Yang Ming Chiao Tung University, Taipei 112, Taiwan; 5 Division of Nuclear Medicine, Department of Radiology, Far Eastern Memorial Hospital, New Taipei City 220, Taiwan; 6 Division of Cardiology, Cardiovascular Medical Center, Far Eastern Memorial Hospital, New Taipei City 220, Taiwan; 7 Medical Device Innovation and Translation Center, National Yang Ming Chiao Tung University, Taipei 112, Taiwan

**Keywords:** breast cancer, volumetric-modulated arc therapy, helical tomotherapy, regional nodal irradiation, left ventricular ejection fraction

## Abstract

Background: Protecting cardiac function in patients with advanced left-breast cancer receiving radiation therapy (RT) with regional nodal irradiation (RNI) is an important issue. Modern RT techniques can limit cardiac exposure. The aim of this study was to explore the association be-tween cardiac dose and cardiac function. Methods: Between 2017 and 2020, we retrospectively reviewed left-breast cancer patients who received adjuvant RT, including RNI with either volumetric-modulated arc therapy (VMAT) or helical tomotherapy (HT). Left ventricular ejection fraction (LVEF) was assessed by echocardiography before RT and 1 year after RT to detect any early deterioration in cardiac systolic function. Results: A total of 30 eligible patients were enrolled. The median follow-up time from the initiation of RT was 3.9 years (range 0.6–5 years). Seventeen patients received VMAT, and the other 13 patients received HT. The median RT dose was 55 Gray (Gy), and the mean heart dose was 3.73 Gy (range 1.95–9.36 Gy). The median LVEF before and after RT was 68% and 68.5%, respectively. No obvious deterioration was found. There was no association between cardiac dose (mean heart dose, V5–V30) and LVEF (change in values or post-RT). Conclusions: For left-breast cancer patients undergoing RT with RNI, VMAT, or HT can be used to limit cardiac exposure. Cardiac function as evaluated by LVEF revealed no obvious deterioration after RT in our patients, and no association was found between cardiac dose and LVEF in those treated with either VMAT or HT in early cardiac surveillance.

## 1. Introduction

The beneficial effect of radiation therapy (RT) on controlling breast cancer disease after breast-conserving surgery or mastectomy and axillary surgery has been documented even with the use of systemic therapy in meta-analyses of randomized trials with long-term follow-up [1,2]. In addition, for breast cancer patients with lymph node (LN) metastases, post-operative RT with regional nodal irradiation (RNI) has been shown to reduce mortality and recurrence [3,4,5]. The use of an extensive RT field will increase the exposure dose to various organs, including the heart, and the reported as-sociation between cardiac dose and cardiovascular disease (CVD) is a concern, especially for patients with left-breast cancer [6,7,8,9]. Moreover, the recent WECARE study reported an association between RT and coronary artery disease (CAD) in young women, and those with left-breast cancer had a more than two-fold higher risk of CAD than those with right-breast cancer [10].

The outcomes of a randomized trial at the University of Michigan for patients with left-breast undergoing RT with RNI demonstrated a reduction in cardiac dose with the use of intensity modulated radiation therapy (IMRT) compared with three-dimensional conformal radiation therapy (3D-CRT). Heart-sparing techniques have also been shown to provide clinically meaningful outcomes in regard to pre-RT and post-RT changes in left ventricular ejection fraction (LVEF) [11]. This highlights the importance of minimizing the cardiac dose to preserve cardiac function. Modern RT techniques such as volumetric-modulated arc therapy (VMAT) and helical tomotherapy (HT) have been shown to be helpful to optimize target coverage and dose conformity; reduce exposure to normal organs, such as the heart; and further improve the therapeutic rate ratio, compared with conventional IMRT or field-in-field techniques for breast cancer patients requiring RT, including RNI [12,13,14,15]. However, even with modern RT techniques, the cardiac mortality rate is proportional to the mean heart dose [16].

To date, the constraints recommended for the heart have been mainly developed for post-operative whole-breast irradiation only. If comprehensive RNI is indicated for left-breast cancer patients, a higher cardiac dose would be expected, for which optimal recommended constraints are lacking. Darby et al. reported a proportional increase in the relative risk of major cardiac events and mean heart dose of 7.4% per Gray (Gy), and that the greatest increase in the rate of major coronary events was within the first 4 years after radiation exposure at 16.3% per Gy, compared with 8.2% ≥ 20 years [17].

Concerns over the cardiotoxicity of left-breast RT, especially in those indicated for extensive RNI, has increased interest in exploring the potential benefits of modern arc techniques with regard to improving dosimetry parameters, reducing heart exposure, and minimizing the impairment of cardiac function. Therefore, the aim of this study was to assess the association between cardiac dose and cardiac function, as evaluated by changes in LVEF before and 1 year after treatment.

## 2. Materials and Methods

### 2.1. Patient Population and Cardiac Function Surveillance

Between January 2017 and March 2020, we reviewed left-breast cancer patients who received adjuvant RT at Far Eastern Memorial Hospital (FEMH). The inclusion criteria were patients (1) with pathologically diagnosed breast cancer; (2) who had undergone lumpectomy or mastectomy surgery; (3) who had received post-operative RT with RNI (optional internal mammary nodes), using modern arc techniques with either VMAT or HT; and (4) with cardiac function data, as assessed by echocardiography, before and after RT. The exclusion criteria were patients (1) with bilateral breast cancer, (2) who received breast or chest wall RT alone without RNI, and (3) without comprehensive echocardiography data.

All patients underwent echocardiography for cardiac risk stratification, screening for any underlying CVD or heart dysfunction, and assessments for cardiac risk before performing RT. LVEF measured by echocardiography was used to assess left ventricular (LV) systolic function. If the patients had CVD, cardiac dysfunction, LVEF ≤ 50%, or any symptoms of heart failure (HF), then they received management from a cardiologist before further cancer therapy. Follow-up echocardiography was arranged 1 year after completing RT to detect any deterioration in LVEF and to any early cardiac dysfunction. The change in LVEF was defined as the difference between post-RT and pre-RT examinations. This study was approved by the Human Experimentation Committee of Far Eastern Memorial Hospital (FEMH-109107-F).

### 2.2. RT Treatment Plan

The patients were placed in the supine position and allowed to breathe freely during RT treatment. The clinical tumor volume (CTV) included the whole breast or chest wall, supraclavicular and infraclavicular regions, any part of the axillary bed at risk, and optional internal mammary nodes for RNI. The planning target volume (PTV) was defined as the CTV plus a 5–8 mm margin to allow for setup errors. The RT prescription was a conventional dose of 45–50.4 Gy in 25–28 fractions, or a hypofractionated dose of 40–42.5 Gy in 15–16 fractions with a daily fraction to the breast or chest wall and RNI. An additional 10–16 Gy boost dose to the tumor bed or surgical scar was allowed. If there were grossly involved or enlarged unoperated LNs, an additional RT boost could be delivered.

### 2.3. RT Techniques and Dosimetry Evaluation

VMAT or HT was planned with the aim of accomplishing better homogeneity and conformity of target coverage while sparing adjacent normal organs to minimize exposure to the heart and other organs at risk. A dose-volume histogram was used to evaluate the dose constraints of the target volumes and other organs at risk. The criteria were at least 100% of the CTV was to receive 100% of the prescribed dose, at least 95% of the PTV was to receive 95–100% of the prescribed dose, and the maximal dose to the PTV region should be less than 110% of the prescribed dose. The constraints to the heart were a mean heart dose < 20 Gy, and V25 < 10%. A Pinnacle3 planning system (version 9.8.1, Philips Medical Systems, Madison, WI, USA) was used to plan VMAT, which was delivered by using a linear accelerator machine (Versa HDTM, Elekta, Crawley, West Sussex, UK). A tomotherapy Hi Art Planning system (version 5.1.3, Tomotherapy, Inc., Madison, WI, USA) was used to plan HT, which was delivered by using a Tomotherapy^®^ Hi-Art or HD system (Tomotherapy^®^; Accuray Inc., Madison, WI, USA).

### 2.4. Statistical Analysis

We used Pearson correlation coefficients to measure the association between cardiac dosimetry factors (heart mean dose, V5–V30) and LVEF (pre-RT, post-RT, or change in value). The degree of correlation was defined by the coefficient values (Pearson r values). It was defined as perfect correlation if the coefficient value was near ±1, strong correlation if the value was between ±0.50 and ±1, medium correlation if the value was between ±0.30 and ±0.49, weak correlation if the value was below ±0.29, and no correlation if the value was zero. SPSS software version 28.0 (SPSS Inc., Chicago, IL, USA) was used for all statistical analyses.

## 3. Results

### 3.1. Demographics

**Figure 1:**
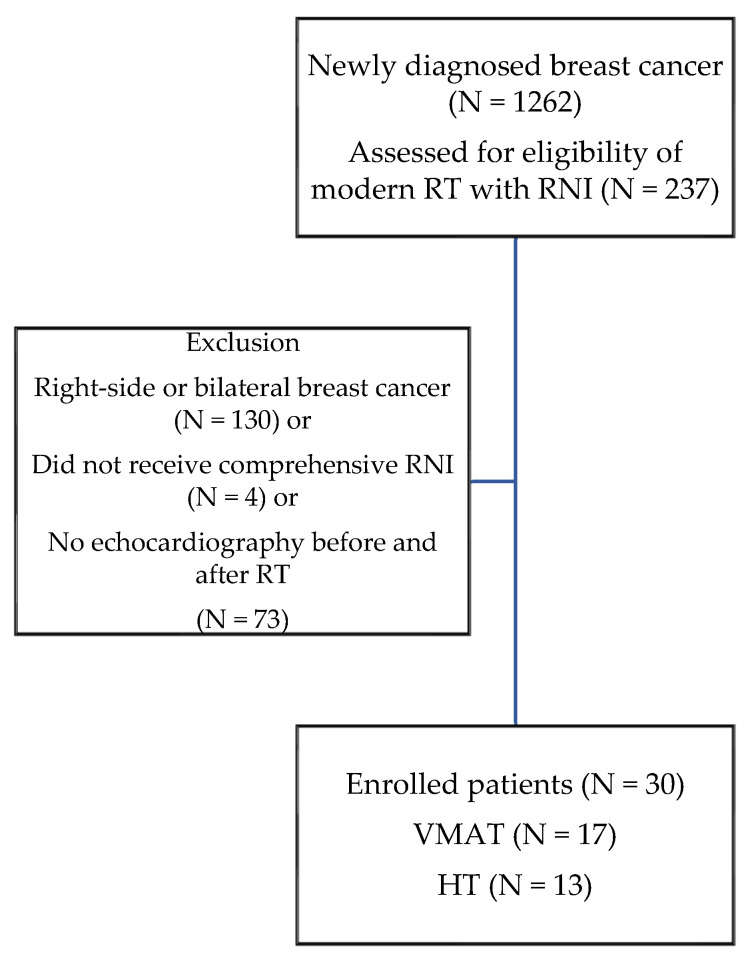
The process of patient enrollment. Abbreviations: RT—radiation therapy; RNI—regional nodal irradiation; VMAT—volumetric modulated arc therapy; HT—helical tomotherapy.

From January 2017 to March 2020, there were 1262 newly diagnosed breast cancer patients at our institution. Among them, 237 patients received curative post-operative RT with RNI, using modern VMAT or HT techniques, and 107 had left-breast cancer. After excluding patients who did not receive comprehensive RNI and those without cardiac function data before and after RT, a total of 30 eligible patients were enrolled in this study. The process of patient enrollment is shown in Figure 1. The median follow-up time from the initiation of RT was 3.9 years (range 0.6–5 years). All of the patients completed RT with RNI. Seventeen patients received VMAT, and the other 13 patients received HT. The median RT dose was 55 Gy (range 43.5–70 Gy). The median age of the patients was 57.5 years (range 39–80 years), and their median body mass index was 25.3 kg/m^2^ (range 17.4–37.9 kg/m^2^). Three (10%) patients had preexisting CVD, including one with HF, one with CAD, and one with both underlying diseases. Twelve (40%), nine (30%), nineteen (63.3%), and three (10%) patients had associated cardiovascular (CV) risk factors of hypertension, diabetes mellitus, dyslipidemia, and smoking. The median LVEF on baseline echocardiography before RT was 68% (range 41–79%). Twenty-four of the patients received anthracycline chemotherapy, and eleven received anti-human epi-dermal growth factor receptor-2 (HER-2/ErbB2) target therapy before irradiation. Details of the patients’ characteristics are presented in Table 1.

### 3.2. RT Dosimetry Outcomes

The mean heart dose in all patients was 3.73 Gy (standard deviation (SD) 1.9, range 1.95–9.36 Gy). Even in the patients who received left-breast RT with RNI, cardiac sparing was excellent with both VMAT and HT. The mean heart dose was lower than that re-ported in a systematic review of modern RT, including 647 regimens published from 2010 to 2015 (average heart dose, 4.4 Gy) [16]. The cardiac dose distributions of V5, V10, V15, V20, V25, and V30 among the 30 patients were 13.5, 6.6, 4.5, 2.25, 1.15, and 0.5%, respectively. Details of RT treatment factors and dosimetry are presented in Table 2.

### 3.3. Cardiac Dose and Cardiac Function

**Figure 2:**
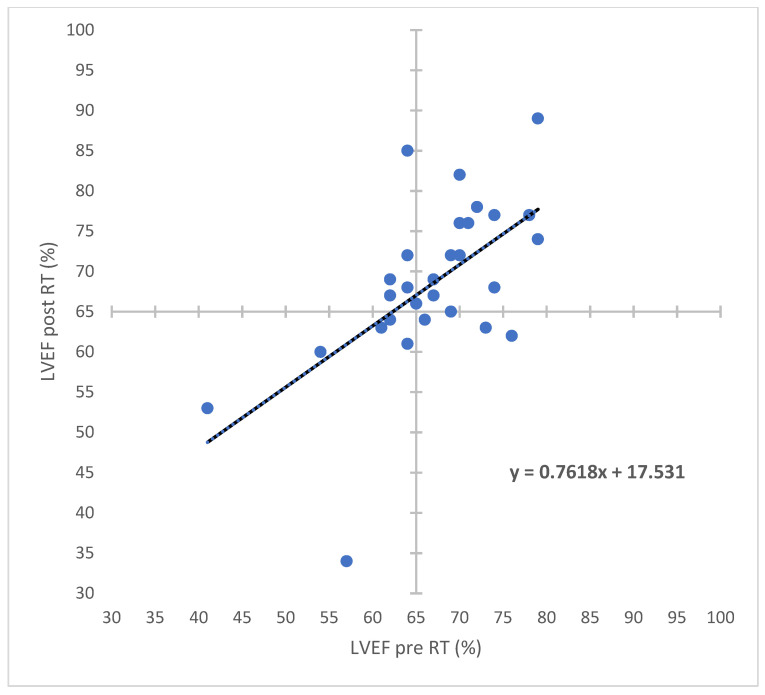
The assessment of LVEF before and after RT for the 30 eligible left-breast cancer patients who received RNI with VMAT or HT. The linear function is included. Abbreviations: LVEF—left ventricular ejection fraction; RT—radiation therapy; RNI—regional nodal irradiation; VMAT—volumetric-modulated arc therapy; HT—helical tomotherapy.

The median LVEF before RT was 68% (SD 8.17, range 41–79%). The median interval between pre-RT and post-RT echocardiography examinations was 1.5 years (range 0.6–3.7 years). The median LVEF post-RT was 68.5% (SD 10.24, range 34–89%), and the change in LVEF between post-RT and pre-RT was 2% (range −23–21%). The assessment of LVEF before and after RT in the 30 patients using quadrant bisectors is shown in Figure 2. The images of LVEF measured by echocardiography before and after RT are shown in Figure S1a,b. No obvious deterioration was found on echocardiography, and there were no associations between cardiac dose (heart mean dose, V5–V30) and LVEF (change in values or post-RT). Pearson correlation coefficients are shown in Table 3. Although there were no significant correlations between heart dose and LVEF, we found relatively higher ab-solute r values for post-RT LVEF (0.213, 0.230, and 0.197) than for LVEF_change (0.171, 0.175, and 0.174) with respect to heart V10, V15, and V20, respectively. That is, the LVEF after RT had a greater probability of being correlated with heart exposure than LVEF_change.

## 4. Discussion

In the era of RNI to improve disease-free survival and breast cancer mortality [1,2], cardiac sparing is an important issue, and cardiotoxicity should be considered. In this paper, we reported the cardiac sparing outcomes of modern rotational RT techniques for locally advanced left-side breast cancer patients. The mean heart dose was reduced with both VMAT and HT in the patients who underwent comprehensive RNI to achieve better cancer-specific survival. In addition, the mean heart dose of 3.73 Gy is lower than published data for traditional techniques, and a lower dose will reduce treatment-related cardiac adverse effects. This was shown by our cardiac function findings, which showed no obvious deterioration in LVEF after RT and no change in LVEF post-RT and pre-RT. Suggested constraints for the heart are currently lacking for advanced breast cancer patients requiring RNI; therefore, investigating the relationship between heart exposure and cardiac function impairment is helpful to clarify and further define recommendations for heart sparing in RT planning and clinical practice.

In the present study, we found no associations between cardiac dose parameters and LVEF. The relatively higher correlation coefficient for post-RT LVEF compared to LVEF_change with respect to heart dosimetry factors may suggest a direction for further studies. The median follow-up time of 3.9 years (range 0.6–5 years) from the initiation of RT in this study is relatively short regarding some CVDs after heart irradiation. Darby et al. reported that the greatest increase in rate of major coronary events was 16.3% per Gy 0–4 years after radiation exposure, more than 1.2% in 10–19 years, and 8.2% ≥ 20 years [17]. Most of the patients in our study received cardiotoxic agents before initiating RT, including anthracycline chemotherapy (80%) or anti-ErbB2 target therapy (36.7%). The early onset of chronic cardiotoxicity within 1 year after completing anthracycline or anti-ErbB2 target therapy, or acute cardiotoxicity manifesting as LV dysfunction has been reported in several studies [18,19,20,21,22,23]. For breast cancer patients undergoing different types of therapy to control the disease, a cumulative effect on cardiac impairment may be expected. Therefore, the early detection of cardiac dysfunction after RT is helpful to allow for aggressive CV management and avoid severe CV morbidity and mortality.

A meta-analysis of 13,000 women in 14 trials by the Early Breast Cancer Trialists’ Collaborative Group revealed that, for patients receiving RNI and treatment with traditional techniques before the 1980s, the mean heart dose was estimated to be >8 Gy, compared with <8 Gy in studies conducted from 1990 to 2000 [24]. A more recent prospective study from the Memorial Sloan-Kettering Cancer Center in 2010s reported an average mean heart dose of 13.2 Gy (range 8.6–20 Gy) for patients with left-breast cancer receiving RNI with an inverse-planned multibeam IMRT technique [25]. VMAT has been reported to achieve a lower mean heart dose than IMRT and 3D-CRT under free-breathing status in several dosimetry comparison studies about different RNI techniques in patients with left-breast cancer [26,27]. In cases where an extensive RT volume is indicated, modern RT techniques have a role in protecting normal organs and avoiding heart impairment. VMAT is the favored technique in patients receiving left-breast RT with RNI, as demonstrated in our previous report [28].

Advances in breast cancer treatment, including multiple modalities of local therapy and systemic therapy, have reduced the cancer mortality rate. Anthracycline-based chemotherapy, anti-ErbB2 target therapy, and breast RT are commonly used for patients with breast cancer to prolong survival. However, cardiac impairment and CV morbidity related to these cardiotoxic agents have also been reported, especially among long-term cancer survivors [17,29,30,31]. LV systolic function is an important predictor of CV outcomes [32,33]. LVEF is the most commonly used parameter used to evaluate LV systolic function for risk stratification and for the management of CVD, and an association between LVEF and mortality has been confirmed. A greater reduction in LVEF is related to a progressive increase in the risk of death or CV hospitalization, and its prognostic value has been demonstrated in a broad range of patients with both cardiac and noncardiac causes [34]. LVEF assessment is helpful to adjust potentially cardiotoxic cancer therapy. There are variable definitions of cardiac dysfunction according to an abnormal or decreased LVEF. In general, cardiotoxic therapy with anthracycline or an-ti-ErbB2 should be withheld in patients with an LVEF < 40%.

The ejection fraction can be measured by using different tools. The most common method is volume-based measurement by echocardiography, which provides timely information and is widely available worldwide. Although it has limitations in detecting some clinical conditions, such as HF with preserved ejection fraction (HFpEF), quantitative LVEF measurements are still the cornerstone of cardiac function evaluation, considering their powerful prognostic prediction ability and extensively utilization [34]. Measurements of myocardial function using global longitudinal strain (GLS) is an alternative to LVEF to assess global LV systolic function. In addition, GLS has been shown to have significant incremental predictive value for mortality in patients with an LVEF > 35% [35].

Baseline CV risk assessments including echocardiography or alternative cardiac imaging modalities are recommended for patients receiving potentially cardiotoxic cancer therapies. The American Society of Clinical Oncology Practice guidelines state that breast cancer patients who receive chest RT ≥ 30 Gy or a lower dose of RT (<30 Gy) in combination with anthracycline treatment are at an increased risk of developing cardiac dysfunction [36]. The European Society for Medical Oncology consensus also highlights therapy-related CV toxicity in breast cancer patients receiving specific chemotherapy, anti-ErbB2 target therapy, and left-sided chest RT [37]. Echocardiography is the preferred tool to evaluate CV condition before, during, and after cancer therapy in patients at risk of cardiac dysfunction. Furthermore, the consensus recommends that, after completing cancer treatment, regular follow-up cardiac echocardiography should be performed every 6 to 12 months.

However, in another study investigating cardiotoxicity during long-term trastuzumab treatment in breast cancer patients, Bouwer et al. found that, in non-smoking patients with baseline LVEF > 60% and without cardiotoxicity during trastuzumab treatment, serial cardiac monitoring could be omitted because of the low cumulative incidence of severe cardiotoxicity [38]. The heart sparing effect with modern RT techniques is promising, and the frequency of CV surveillance can be modified according to individual risk stratification. In selected low-risk patients, the future development of precision medicine is expected to decrease the frequency or omit long-term CV monitoring. Based on this preliminary report of the effect of VMAT or HT on cardiac function, further studies with more patients using modern RT techniques to reduce cardiac exposure and longer follow-up period for cardiac function are warranted to help define more precise risk stratification of cardiotoxicities.

There are limitations to this study. It is a retrospective study performed at a single institution, and with a relatively short follow-up period considering the development of cardiotoxicities after RT which have been reported to occur from a few weeks to several decades [39]. Longer surveillance will therefore be necessary to detect late cardiac events. Most of the enrolled patients did not have preexisting CVD, and most received chemotherapy before RT. However, the small number of patients and their heterogenous characteristics and baseline CV risk factors limit our analysis of the relationship between heart dose and cardiac function. Most patients received conventional fractionation rather than hypofractionation in this study. Although the conventional schedule is the current standard recommendation for patients receiving RNI, hypofractionation or ultrahypofractionation regimens should also be considered, as they may be more common in the future. We used only LVEF, as assessed by standard echocardiography, to evaluate cardiac function in this study. GLS that can detect and quantify subclinical and subtle disturbances in LV systolic function, and it may have a role as an early marker. Other cardiac biomarkers such as troponin and N-terminal pro-brain natriuretic peptide (NT-proBNP), and alternative cardiac imaging modalities such as myocardial perfusion imaging and magnetic resonance imaging can provide highly sensitive and specific information of cardiotoxicity.

This is a preliminary report of cardiac function after modern RT in advanced left-breast cancer patients, and our results confirmed an association between heart dose and cardiac function. Early adverse cardiac effects were limited in these patients who were treated with modern rotational RT techniques, even with an extensive radiation volume. A further analysis of integration with strain echocardiography, serum cardiac biomarkers, myocardial perfusion imaging, cardiac magnetic resonance imaging, or other cardiac surveillance tools will provide more comprehensive cardiac function evaluation. A longer follow-up period to detect late cardiotoxicities will be helpful to clarify the relationship between cardiac exposure and cardiac function in future studies.

## 5. Conclusions

Multimodality therapy and a multidisciplinary approach are crucial in breast cancer treatment to maximize disease control and minimize therapy-related side effects. For left-breast cancer patients undergoing RT with comprehensive RNI, modern RT techniques with VMAT or HT can limit cardiac exposure. The mean heart dose in this study was 3.73 Gy, which is lower than in a previous systematic review. Cardiac function evaluated according to LVEF after RT or deterioration in LVEF revealed no obvious impairment. No associations were found between cardiac dose parameters (heart mean dose, V5–V30) and LVEF (change in values or post-RT) in early cardiac surveillance. Further studies are warranted to investigate whether post-RT LVEF is correlated with heart exposure.

## Figures and Tables

**Table 1:** Patient characteristics.

Characteristics		Characteristics	
Age (years) (SD) (range)	57.5 (11) (39–80)	Smoking: N (%)	3 (10)
BMI (kg/m^2^) (SD) (range)	25.3 (5.24) (17.4–37.9)	Chemotherapy before RT: N (%)	29 (96.7)
Baseline LVEF (%) (SD) (range)	68 (8.17) (41–79)	Anthracycline-containing chemotherapy regimen: N (%)	24 (80)
Cardiac disease: N (%)	3 (10)	Taxane-containing chemotherapy regimen: N (%)	21 (70)
Diabetes mellitus: N (%)	9 (30)	Anti HER-2/ErbB2 target therapy: N (%)	11 (36.7)
Hypertension: N (%)	12 (40)	Aromatase inhibitor therapy: N (%)	12 (40)
Dyslipidemia: N (%)	19 (63.3)	Tamoxifen therapy: N (%)	14 (46.7)

Abbreviations: SD—standard deviation; BMI—body mass index; LVEF—left ventricular ejection fraction; HER-2/ErbB2—human epidermal growth factor receptor-2.

**Table 2:** RT treatment factors and dosimetry.

RT Treatment Factors		RT Dosimetry	Median
VMAT: N (%) HT: N (%)	17 (56.7) 13 (43.3)	RT total dose (Gy) (SD) (range)	55 (5.73) (43.5–70)
Lumpectomy: N (%) Mastectomy: N (%)	15 (50%) 15 (50%)	Number of fractions (SD) (range)	30 (4.34) (15–35)
Conventional fractionation: N (%) Hypofractionation: N (%)	28 (93.3%) 2 (6.7%)	Heart Volume (SD) (range)	489.5 (115.7) (356.5–947)
CTV (mL) (SD) (range)	450 (392.2) (158.5–1845.6)	Mean heart dose (Gy) (SD) (range)	3.73 (1.9) (1.95–9.36)
PTV (mL) (SD) (range)	892.9 (574.9) (402.2–2937.1)	V5 (%)	13.5
**RT target volume**		V10 (%)	6.6
Breast_SCF	9 (30%)	V15 (%)	4.5
Breast_SCF_IMN	6 (20%)	V20 (%)	2.25
CW_SCF	6 (20%)	V25 (%)	1.15
CW_SCF_IMN	9 (30%)	V30 (%)	0.5

Abbreviations: RT—radiation therapy; VMAT—volumetric-modulated arc therapy; HT—helical tomotherapy; CTV—clinical tumor volume; SD—standard deviation; PTV—planning target volume; SCF—supraclavicular fossa; IMN—internal mammary nodes; CW—chest wall; Gy—Gray.

**Table 3:** Associations between cardiac dose (heart mean dose, V5–V30) and LVEF (pre-/post-RT, change in values) in the 30 eligible left-breast cancer patients. The coefficient values were calculated using the Pearson correlation coefficient method.

	LVEF_Change	LVEF Pre-RT	LVEF Post-RT	Heart Mean Dose	Heart V5	Heart V10	Heart V15	Heart V20	Heart V25	Heart V30
LVEF_Change	1									
LVEF pre-RT	−0.233	1								
LVEF post-RT	0.631 ***	0.608 ***	1							
Heart Mean	−0.125	0.009	−0.095	1						
Heart V5	−0.135	0.014	−0.099	0.942 ***	1					
Heart V10	−0.171	−0.092	−0.213	0.963 ***	0.917 ***	1				
Heart V15	−0.175	−0.109	−0.230	0.915 ***	0.773 ***	0.949 ***	1			
Heart V20	−0.174	−0.068	−0.197	0.859 ***	0.670 ***	0.878 ***	0.977 ***	1		
Heart V25	−0.114	−0.023	−0.111	0.763 ***	0.541 **	0.770 ***	0.909 ***	0.962 ***	1	
Heart V30	−0.059	−0.017	−0.062	0.658 ***	0.425 *	0.626 ***	0.781 ***	0.862 ***	0.938 ***	1

* *p* < 0.05, ** *p* < 0.01 and *** *p* < 0.001. Abbreviations: LVEF—left ventricular ejection fraction; RT—radiation therapy.

## Data Availability

The data presented in this study are available upon request from the corresponding author. The data are not publicly available due to patients’ privacy and medical ethics.

## Supplementary Materials

The images of LVEF measured by echocardiography before and after RT. The following are available online at https://www.mdpi.com/article/10.3390/bioengineering9050213/s1. Figure S1: The LVEF measured by echocardiography before and after RT for a left-breast cancer patient.
