# Supplementary figures and images for: Cardiac Function after Modern Radiation Therapy with Volumetric Modulated Arc Therapy or Helical Tomotherapy for Advanced Left-Breast Cancer Receiving Regional Nodal Irradiation
Source: Bioengineering (Basel). 2022 May 16;9(5):213. doi: 10.3390/bioengineering9050213 (PMC9138009; doi:10.3390/bioengineering9050213)

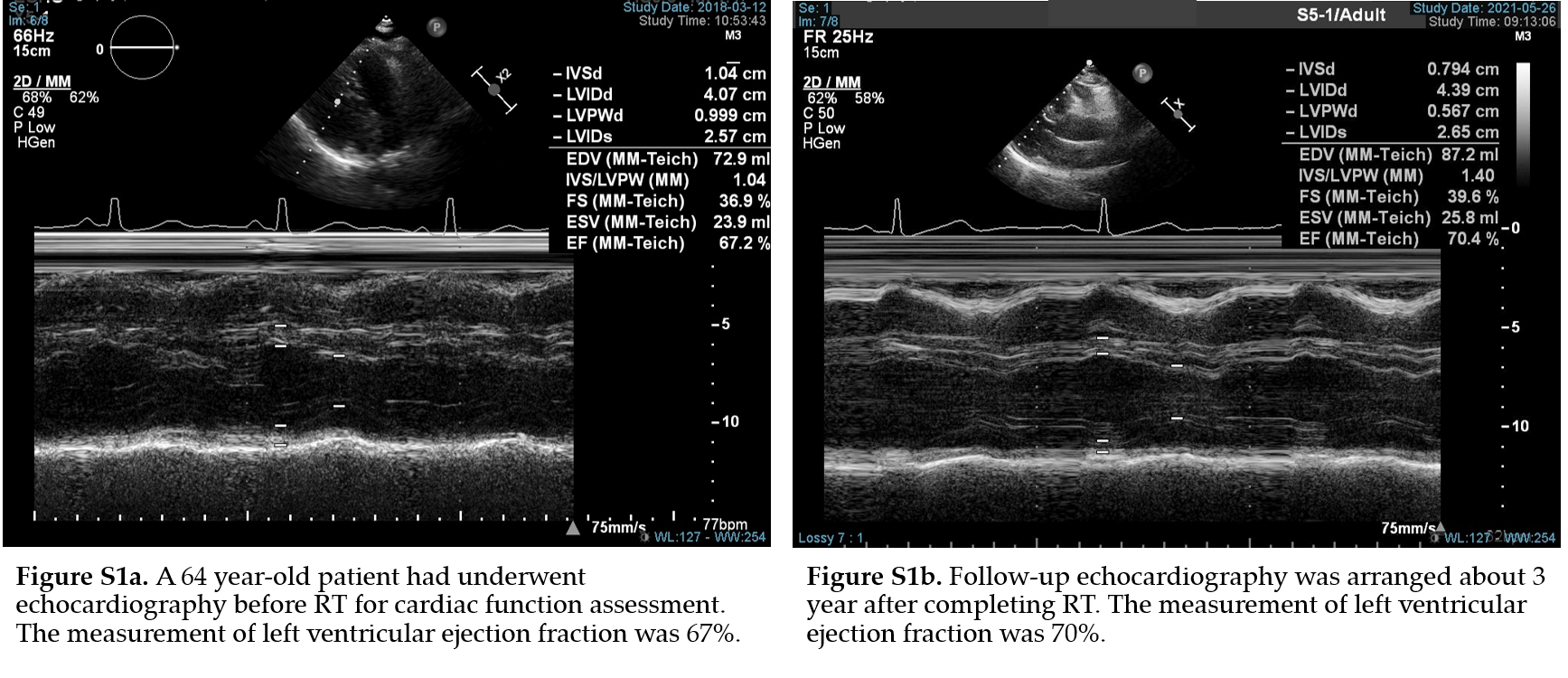

Supplement: Supplementary file 1 [file bioengineering-09-00213-s001.zip › Figure S1ab.png]
